# Drug-Induced Liver Injury Associated with the Use of Everolimus in a Liver Transplant Patient

**DOI:** 10.1155/2018/7410508

**Published:** 2018-07-18

**Authors:** Serena Patel, Michel H. Mendler, Mark A. Valasek, Shirley M. Tsunoda

**Affiliations:** ^1^University of California (UC) San Diego, Skaggs School of Pharmacy and Pharmaceutical Sciences, USA; ^2^UC San Diego, Department of Medicine, Division of Hepatology, USA; ^3^UC San Diego, Department of Pathology, USA

## Abstract

Drug-induced liver injury (DILI) has not been previously reported as a complication of treatment with everolimus. A 56-year-old Caucasian male liver transplant recipient developed DILI after receiving everolimus. Elevations in transaminase levels occurred within a week of starting everolimus and an upward trend in the transaminase levels continued with supporting histopathologic changes confirmed by liver biopsy. Within one week of drug discontinuation, his liver enzymes normalized to baseline. This report includes a brief review of the pharmacokinetic properties of everolimus, a review of the relevant literature, and an analysis using the RUCAM and Naranjo algorithms.

## 1. Introduction

Everolimus is a mammalian target of rapamycin (mTOR) inhibitor that is FDA approved for use as an immunosuppressive agent in kidney and liver transplantation [1]. In liver transplantation, everolimus is used with low dose tacrolimus and steroids as a kidney-sparing agent to prevent cellular rejection [2]. Everolimus is also approved for advanced cases of breast cancer, pancreatic tumors, and advanced renal cell carcinoma [1]. As a narrow therapeutic index drug, achieving optimal exposure levels (trough concentration ranging from 3 to 8 ng/mL) is critical to its successful use [3].

The mTOR pathway is involved in several physiological pathways including protein, nucleotide, and lipid synthesis. mTOR is a protein kinase that regulates cell growth and cycle progression of B and T lymphocytes from the IL-2 receptor to the nucleus [4]. Consequently, it comes with a diverse range of dose-dependent side effects including hyperlipidemia, edema, wound healing complications, stomatitis, anemia, proteinuria, and interstitial pneumonitis.

In this case, we discuss a patient on everolimus after liver transplant and the histopathologic and laboratory value changes indicative of drug-induced liver injury (DILI).

## 2. Case Presentation

**Figure 1:**
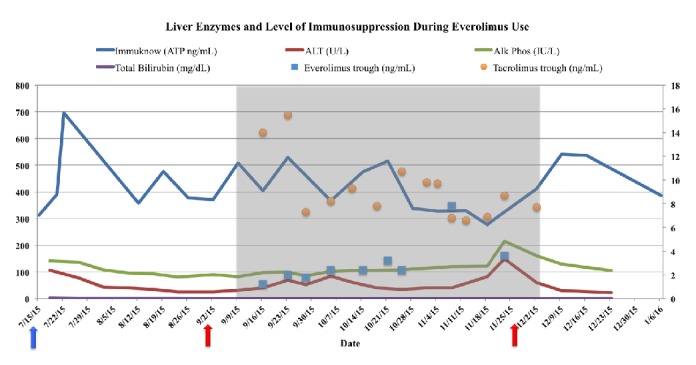
Patient's liver enzyme levels and level of immunosuppression after receiving a liver transplant in July 2015. The blue arrow represents the date of the transplant and the shaded area between the red arrows represents the everolimus exposure period (start: 9/3/15; end: 12/1/15).

A 56-year-old Caucasian male with nonalcoholic steatohepatitis (NASH) experienced progression to cirrhosis and its complications including portal hypertension, esophageal varices, and ascites. He had no other significant past medical history. At the time of transplant in July 2015, the patient weighed 228 lbs (BMI 34). He received a liver transplant and was placed on a maintenance immunosuppressive regimen of tacrolimus 9 mg PO BID with a trough goal of 8-10 ng/mL, mycophenolic acid (Myfortic) 720 mg PO BID, and a prednisone taper. The patient remained stable on this regimen and had the following normal laboratory results at the beginning of September (**Figure 1**): ALT 32 IU/L (normal=0-40), AST 23 IU/L (normal= 5-40), alkaline phosphatase (ALP) 83 IU/L (normal= 40-100), gamma-glutamyl transpeptidase (GGT) 36 IU/L (normal=10-64), total bilirubin 0.3 mg/dL (normal=0.3-1.9 mg/dL), BUN 26 (normal 6-20 mg/dL), and Scr 1.18 (normal 0.67-1.17 mg/dL) and INR 1.1.

On September 3, 2015, the patient was switched from mycophenolic acid to everolimus as part of a clinical research study investigating the renal sparing effects of everolimus to target a lower tacrolimus trough concentration of 3-5 ng/mL. At the time of everolimus introduction, the patient's weight was down to 210 lbs (BMI 31.9) and laboratory values that would impact the pharmacokinetics of everolimus were within a normal range: Hgb 12 mg/dL and albumin 3.7 g/dL. After the patient's first everolimus dose on a starting regimen of 1 mg PO BID, he reported new onset pain to the right flank area. At this time, there was an upward trend in his liver enzymes, ALT (69 IU/L), AST (35 IU/L), ALP (99 IU/L), and GGT (58 IU/L). The everolimus trough was subtherapeutic until late October when a trough of 3.8 ng/mL was achieved on a dose of 3 mg PO qam and 2.5 mg PO qpm (**Figure 1**).

In early October, the patient experienced increasing liver enzymes (ALT=84 IU/L; AST= 42 IU/L; ALP=102 IU/L; GGT=53 IU/L; total bilirubin 0.23 mg/dL) with a tacrolimus trough concentration of 8.2, so a liver biopsy was performed to rule out rejection. The results showed mild portal inflammation with lymphocytes, pericentral sinusoidal dilatation with no hepatic plate atrophy, and inflammation adjacent to the central vein (RAI score = 1 out of 9). The trichrome stain did not have any perisinusoidal staining to indicate chronicity, nor was there duct injury, duct loss, cholestasis, endothelitis, or steatosis. Immunostains for hepatitis B surface antigen (HBsAg) and hepatitis B core antigen (HBcAg) were also negative. There was no report of fever, chills, dark-colored urine, or jaundice or any evidence of an acute hypersensitivity reaction (fever/rash). The immune cell function (ImmuKnow) assay was 338 ng/mL in late October close to the time of the biopsy (10/26/15). It was confirmed that there was no evidence of acute rejection.

**Figure 2:**
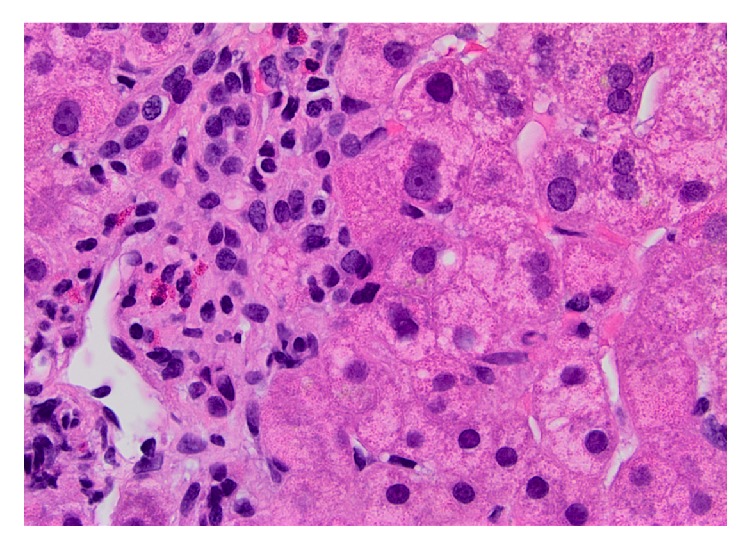
Patient's second biopsy done on 11/30/2015 showing features of DILI including portal eosinophils and rare acidophil bodies.

By the end of November, two and a half months from the start of everolimus, the patient's serum liver enzymes reached their highest values, ALT (149 IU/L), AST (81 IU/L), and ALP, (215 IU/L) and the ImmuKnow assay done at this time resulted in a level of 412 ng/mL. Tacrolimus doses had not been changed and troughs ranged from 6.9 to 9.8 ng/mL. A second liver biopsy was done on 11/30/15 that showed mild patchy sinusoidal dilatation and focal mild inflammatory infiltrate with lymphocytes, eosinophils, and rare acidophil bodies (**Figure 2**). There was no evidence of acute cellular rejection (RAI score= 1 out of 9), but the presence of eosinophils and focal mild portal inflammation was consistent with the possibility of drug injury. Everolimus was discontinued on December 1, 2015, and the patient went back to a regimen of tacrolimus 5 mg BID and mycophenolic acid 720 mg BID. After one week, the liver enzymes returned to normal: ALT (22 IU/L), AST (20 IU/L), ALP (105 IU/L), and total bilirubin (0.6 mg/dL). Since discontinuation, the patient denied pain and dizziness and reported improved energy.

## 3. Discussion

To our knowledge, this is the first report of everolimus associated DILI. A DILI score model was created to better predict the potential of a drug to cause liver injury and the severity of the injury based on the lipophilicity of the drug (log⁡P ≥3), covalent binding of reactive metabolites, and the daily dose of the oral medication (≥ 100 mg) [5]. Everolimus has not been studied in this model directly. However, it does have the property of high lipophilicity with a log⁡P of 5.01, a property associated with greater rates of mitochondrial toxicity and DILI [5, 6]. Secondly, as opposed to sirolimus, it has a stable 2-hydroxy-ethyl substitution at position 40, improving its oral bioavailability [7, 8]. In our patient, the daily dose of everolimus never exceeded 5.5 mg daily, far lower than the 100 mg threshold for the model. The importance of this property for a drug like everolimus may not be significant because there is a poor correlation between the dose and actual systemic exposure of the drug; a stronger correlation is seen between the AUC and trough concentration [7].

Despite the similarity in chemical structures between sirolimus and everolimus, only sirolimus has been included in the FDA's Liver Toxicity Knowledge Base (LTKB) Database as being of “less DILI concern.” This is based on both drug-labeling studies of sirolimus and verified causality evidence of hepatotoxicity with elevated trough levels [9–11]. A reference drug list for risk of DILI in humans ranks everolimus as having “ambiguous DILI concern” when taking into account the drug-labeling studies as well as verified causality evidence [9]. Based on the clinical trial studies, everolimus was initially ranked as having “less DILI concern” due to ≤ 10% of patients in each test group having elevations in alkaline phosphatase and liver transaminases [10]. However, the verified DILI concern is still “ambiguous” because there have been no verified causality evidence reports on DILI [9].

In order to rule out other causalities for DILI in this patient, we examined the DILI potential of his concomitant medications. These included tacrolimus, prednisone, sulfamethoxazole/trimethoprim (TMP-SMZ), metformin, glipizide, and propranolol. Tacrolimus, prednisone, and TMP-SMZ are not listed as drugs with a significant risk of DILI in humans based on the reference compiled by Chen et al. [9]. In postmarketing reports, tacrolimus has only been associated with mild to moderate elevations in serum aminotransferases in 5-10% of patients and any elevations are mild, asymptomatic, and self-limiting [12]. Long term and high doses of prednisone and methylprednisolone (500 mg) [13] have been associated with NASH exacerbations, with liver enzyme elevations, and with liver histology showing hepatitis with steatosis, chronic inflammation, and Mallory bodies, which were not consistent with liver biopsy in this patient [11]. Our patient was only on a small oral maintenance dose of 2.5 mg daily. TMP-SMZ can cause mild elevations in ALT that do not proceed to more severe liver injury or jaundice, but in postmarketing reports, the onset is usually within a few days or weeks of starting the medication and resolves within 2-4 weeks [11]. Though idiosyncratic liver injury has been reported with TMP-SMZ with features of drug allergies (eosinophilia), the time course of onset was not consistent with the literature [11]. In our case, TMP-SMZ was started in July 2015 and the patient's liver enzymes (as evident in the graph) were still stable and within normal limits at this time prior to the start of everolimus in September 2015.

Propranolol has been listed as a drug of “ambiguous DILI concern” by Chen et al. and has been rated as an unlikely cause of clinically apparent liver injury. Its rating is based on case reports showing a pattern of serum enzyme elevations that are hepatocellular in nature with typical onset of 2 to 24 weeks [9, 11]. These elevations have been seen in 2% of patients using propranolol and are transient and asymptomatic and tend to resolve even as the patient continues the medication [11]. There are no causal associations because these case reports included patients who were on other well-known hepatotoxic drugs [11]. This patient had started propranolol on 8/28/15, so while the timecourse of propranolol use may be in conjunction with the time for enzyme elevation with its use, the pattern of enzyme elevation was not hepatocellular.

Though metformin was initially listed as a drug with “no-DILI concern,” there have been close to twenty case reports in the literature of hepatotoxicity with metformin after 4 to 8 weeks of use [14]. Minor elevations in liver enzymes have been reported to occur during metformin therapy in less than 1% of patients and typically the timing of the injury occurs soon after the agent is started, not during long term therapy [11]. Our patient had been taking metformin for three months, so the likelihood of metformin causing his DILI is low. Glipizide has not been identified as an agent of DILI concern and minor elevations in liver enzymes occur in <1% of patients which is comparable to what can be expected with placebo [9, 11]. Though these medications were started after transplant, the timecourse of transaminase elevations does not correspond to the literature reports.

Additionally, we checked to ensure that elevations in liver enzymes and liver injury were not due to drug interactions. The only major interactions noted were between amlodipine (a CYP3A4 inhibitor) and tacrolimus, leading to increased levels, and prednisone (a CYP3A4 inducer) and tacrolimus leading to decreased levels and potential for transplant rejection [15]. Tacrolimus trough levels were monitored throughout this patient's treatment and were within the trough goal throughout the time of liver injury. Therefore, it is unlikely that drug-drug interactions played a role.

The classification of DILI based on phenotype appears to be mixed [11]. At the peak of the patient's enzyme elevations, the ratio of [ALT/ULN]/[Alk P/ULN] was 2.23. This was based on the patient's enzymes measured on 11/23/15 in which the ALT was 149 U/L (ULN= 40) and the Alk Phos was 215 IU/L (ULN= 129) [11, 16]. Ratios of >5 define hepatocellular, <2 cholestatic, and between 2 and 5 mixed pattern of enzymes [11, 16]. In hepatocellular injury, the ALT is usually >three times the upper limit of normal (ULN), alkaline phosphatase is more than two times the ULN, or total bilirubin is more than twice the ULN [11, 16]. Though elevations to this extent were not evident within the first month, the values did increase and eventually reached levels of ALT or AST that were >3X the ULN and an ALP > 2X the ULN by the end of two months. This indicates that the injury may be mixed hepatocellular and cholestatic.

**Figure 3:**
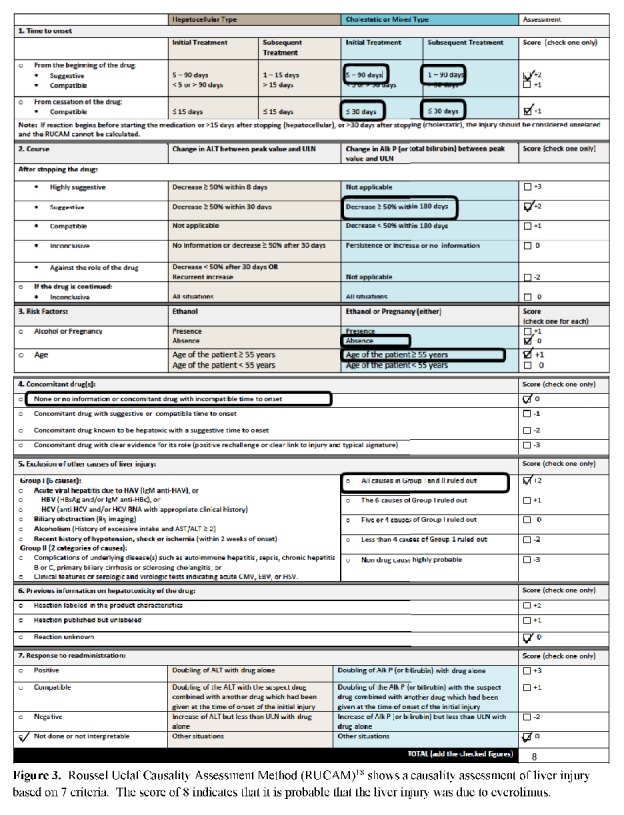
Roussel Uclaf Causality Assessment Method (RUCAM) [16] shows a causality assessment of liver injury based on 7 criteria. The score of 8 indicates that it is probable that the liver injury was due to everolimus.

We suspected everolimus induced DILI based on the chronological association with liver enzyme abnormalities, the pathology findings, and the properties of the drug. We also applied the more standardized causality assessment for drug-induced hepatotoxicity, RUCAM (Roussel Uclaf Causality Assessment Method). This method evaluates drug-induced liver injury based on seven criteria: (1) time to onset of the injury following start of the drug; (2) subsequent course of injury after stopping the drug (time to enzyme normalization after cessation of drug); (3) specific risk factors (age, alcohol use, and pregnancy); (4) use of other medications with a potential for liver injury; (5) exclusion of other causes of liver disease; (6) known potential for hepatotoxicity of the implicated drug; and (7) response to rechallenge [16]. This data is scored and categorized as highly probable, probable, possible, or excluded. RUCAM is specific to liver injury and has been evaluated for accuracy, reproducibility, and intraobserver variability with the advantage of being more objective [11]. The range of possible RUCAM scores is −9 to +14 with 0 or less indicating that the drug is “excluded” as a cause, 1 to 2 indicating that it is “unlikely,” 3 to 5 indicating that it is “possible,” 6 to 8 indicating that it is “probable,” and greater than 8 indicating that it is “highly probable.” In our patient the RUCAM score was 8, which indicates that the link of liver injury as an adverse drug reaction of everolimus is probable (**Figure 3**). While RUCAM is advantageous due to its specificity to liver injury in assessing causality, one drawback is in criterion #6 which asks the observer to consider the known publications of the reaction [16]. The model does not account for differences in type or quantity of publications.

We also applied a second assessment of causality using the Naranjo scale. While the Naranjo scale is not specific for liver injury, it is a widely accepted scale for assessing drug-associated reactions [17]. The assessment takes into consideration ten factors: (1) any previous reports of a reaction; (2) the appearance of the adverse event after starting the drug; (3) improvement in the reaction after drug discontinuation; (4) reappearance of the reaction when the drug is readministered; (5) other possible causes; (6) reappearance of the reaction when placebo is given; (7) detection in the blood or fluids of toxic drug concentrations; (8) worsening or improvement of the reaction with an increase or decrease in dose; (9) previous similar reaction to the drug; and (10) confirmation by objective evidence [17]. The scoring system ranges from −4 to +13 with a score of 0 or less being “doubtful,” 1 to 4 being “possible,” 5 to 8 being “probable,” and 9 or higher being “definite” adverse drug reaction. Based on the Naranjo scale, the likelihood of everolimus causing the liver injury in our patient was 7 (or probable)** (Table 1)**. A probable causality on this scale means that the reaction (liver injury) followed a reasonable temporal sequence after initiation of the drug and was confirmed by withdrawal of the drug.

## 4. Conclusion

This case documents an association between everolimus and DILI. Use of everolimus in this post-liver transplant patient led to elevations in AST, ALT, and ALP consistent with mixed hepatocellular and cholestatic liver injury over a period of three months. In addition, the liver biopsy confirmed the finding of DILI based upon the histological characteristics, notably the presence of eosinophils, rare acidophil bodies, and focal mild portal inflammation. Finally, all other concomitant medications have been sufficiently ruled out as being the cause of DILI. The pharmacokinetic characteristics of everolimus coupled with the temporal correlation, histologic findings, and positive RUCAM and Naranjo scores point to everolimus being the likely cause of this patient's DILI. Future investigations into the factors that may have increased this patient's susceptibility should be explored.

## Figures and Tables

**Table 1:** Adverse drug reaction probability scale (Naranjo) [17].

**Question**	**Yes**	**No**	**Do Not Know**	**Score**
1. Are there previous conclusive reports on this reaction?	+1	0	0	0

2. Did the adverse event appear after the suspected drug was administered?	+2	-1	0	+2

3. Did the adverse event improve when the drug was discontinued or a specific antagonist was administered?	+1	0	0	+1

4. Did the adverse event reappear when the drug was readministered?	+2	-1	0	0

5. Are there alternative causes that could on their own have caused the reaction? (alternatives have been excluded =no)	-1	+2	0	+2

6. Did the reaction reappear when a placebo was given?	-1	+1	0	0

7. Was the drug detected in blood or other fluids in concentrations known to be toxic?	+1	0	0	0

8. Was the reaction more severe when the dose was increased or less severe when the dose was decreased? (reaction was less severe when the dose was decreased)	+1	0	0	+1

9. Did the patient have a similar reaction to the same or similar drugs in any previous exposure?	+1	0	0	0

10. Was the adverse event confirmed by any objective evidence?	+1	0	0	+1

	**Total Score: 7**			

## Abbreviations

CYP3A4: Cytochrome P450 3A4
AUC: Area under the curve
DILI: Drug-induced liver injury
CNI: Calcineurin inhibitor
mTOR: Mammalian target of rapamycin
RUCAM: Roussel Uclaf Causality Assessment Method
NASH: Nonalcoholic steatohepatitis.
